# Blood biomarkers and atrial remodeling in patients at risk of atrial fibrillation

**DOI:** 10.3389/fmmed.2026.1801523

**Published:** 2026-05-22

**Authors:** Filip Loncaric, Ilana Forado Benatar, Maria Mimbrero Guillamon, Loredana Nunno, Rafael Jimenez-Arjona, Laia Tirapu, Sílvia Montserrat, Laura Sanchis, Adelina Doltra, Bart Bijnens, Marta Sitges

**Affiliations:** 1 Klinicki bolnicki centar Zagreb, Zagreb, Croatia; 2 August Pi i Sunyer Biomedical Research Institute (IDIBAPS), Barcelona, Spain; 3 Hospital Clinic de Barcelona, Barcelona, Spain; 4 Institucio Catalana de Recerca i Estudis Avancats, Barcelona, Spain

**Keywords:** athletes, arterial hypertension, atrial remodeling, biomarkers, galectin, speckle-tracking, three-dimensional echocardiography

## Abstract

**Introduction:**

Arterial hypertension and endurance training are related to left atrial (LA) remodeling and risk of atrial fibrillation (AF). Serum blood biomarkers have been explored in relation to cardiovascular risk. Our aim was to assess the influence of patient and echocardiographic characteristics on serum blood biomarkers in individuals at risk for AF.

**Methods:**

A population of 511 subjects was analyzed: 286 endurance athletes and 225 patients with arterial hypertension. Participants underwent 2D and 3D echocardiography with speckle-tracking analysis. Blood samples were obtained to evaluate biomarkers of myocardial damage (Brain Natriuretic Peptide, troponin I, Oncostatin M, heart-type Fatty Acid-Binding Protein and Placenta Growth Factor), vascular endothelial dysfunction (Endothelial cell-specific molecule 1), and fibrosis (Matrix Metalloproteinase 1, 2 and 9, C-X-C motif chemokine ligand 6 and 16, and Galectin-3 (Gal-3)). Linear regression models were fitted to discern the relationship of patient and echo characteristics and serum biomarker levels.

**Results:**

The results revealed the significant influence of age, gender, body mass index and renal function on biomarker levels. Parameters of myocardial damage showed a limited range of values in our cohort with no differences between groups. Biomarkers of fibrosis were uniformly elevated in hypertension, with Gal-3 levels independently associated with LA reservoir strain.

**Conclusion:**

Serum levels of fibrosis biomarkers differ between populations at risk for AF. In hypertension Gal-3 levels are associated with LA reservoir function, making it a potential biomarker for LA remodeling in this setting. Clinical follow-up is needed to relate these findings with clinical outcomes.

## Graphical Abstract

**Figure:**
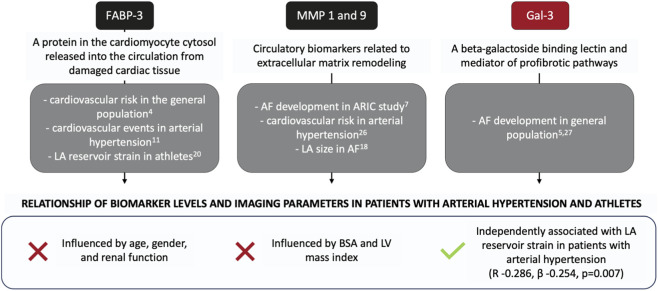
Central Illustration – (First and second row) An overview of selected biomarkers and their association to atrial fibrillation. (Third row) An overview of the study findings regarding various influences of patient characteristics on biomarker levels. Galectin-3 levels were found to have a weak correlation with LA reservoir strain within the hypertensive cohort suggesting a potential role of the biomarker in predicting LA remodeling.

## Introduction

Left atrial (LA) enlargement is commonly seen in arterial hypertension, due to increased atrial pressure related to diastolic dysfunction, and in endurance training, due to volume overload (Trivedi et al., 2020). Pressure and volume loading result in stretching of the atrium and, over time, alterations in the cardiomyocytes, fibroblasts, and non-collagen infiltrative compartments of the atrium (Tsang et al., 2002; De Jong et al., 2011; Goette et al., 2016; Otaki et al., 2014), creating a potential substrate for atrial fibrillation (AF). Serum blood biomarkers related to myocardial damage, endothelial dysfunction and fibrosis have been increasingly explored in relation to cardiovascular risk and therefore evaluated to predict the particular risk of patients for cardiovascular events. (Fashanu et al., 2017; Ionin et al., 2020; Huxley et al., 2013).

Our aim was to assess the influence of patient characteristics and echocardiographic measures of left ventricular (LV) and LA size and function on a comprehensive set of serum blood biomarkers of myocardial injury, endothelial dysfunction and fibrosis in patients at risk for AF.

## Methods

**FIGURE 1:**
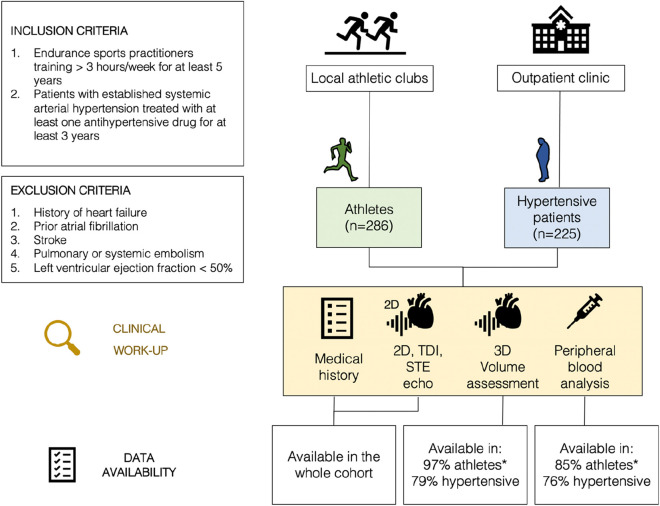
Study overview–Inclusion and exclusion criteria are shown on the left. Data availability shows that hypertensive patients were excluded from the 3D echocardiography protocol due to a poor imaging window (Chi-square p < 0.001). Similarly, hypertensive patients had a lower percentage of adherence to the scheduled peripheral blood draws (Chi-square p = 0.026). (TDI‐Tissue Doppler Imaging, STE‐Speckle Tracking Echocardiography).

The study design was cross-sectional observational with the cohort (n = 511) consisting of two subgroups: endurance sports practitioners (athletes) training more than 3 h per week for at least 5 years (n = 286); and patients with established systemic arterial hypertension treated with at least one antihypertensive drug for at least 3 years (n = 225). The study workflow and inclusion/exclusion criteria are shown in Figure 1. All participants were assessed for cardiovascular risk factors, medical history of target organ damage, comorbidities, and pharmacological treatment; followed by an echocardiographic examination and the collection of a peripheral venous blood sample. The athlete cohort filled out a questionnaire regarding their average weekly training sessions (i.e., type(s) of sport, session duration and intensity) with the aim of estimating the cumulative MET minutes per week for each athlete (Ainsworth et al., 2011). The study was approved by the hospital ethics committees, and participants gave written informed consent.

The echocardiographic examination was performed on a Vivid E95 system (GE, Vingmed Ultrasound, Horten, Norway) equipped with a M5S and 4Vc transducer. Cardiac dimensions were measured in appropriate 2D views and derived parameters calculated, following relevant society recommendations (Lang et al., 2015). Real-time three-dimensional (3D) scans were obtained in the apical view in patients with an appropriate imaging window (in 84% of the cohort, see Figure 1), whereas 3D LA volumes were analyzed using the ‘4D LA quantification tool’ available in GE EchoPAC.

Blood was extracted from a peripheral vein at the same clinical visit as the imaging study. Samples were processed for complete blood count, metabolic panels and lipid profile. Additional blood biomarkers were assessed evaluating myocardial damage (Brain Natriuretic Peptide (BNP); troponin I, Oncostatin M (OSM) (Kubin et al., 2011), serum heart-type Fatty Acid-Binding Protein (FABP3) (Otaki et al., 2014; Setsuta et al., 2014), and Placenta Growth Factor (PIGF) (Draker et al., 2019)), vascular endothelial dysfunction (Endothelial cell-specific molecule 1 (Endocan-1) (Afsar et al., 2014; Çimen et al., 2016)); and fibrosis (Matrix Metalloproteinase 1, 2 and 9 (MMP 1,2,9) (Huxley et al., 2013; Boixel et al., 2003)), C-X-C motif chemokine Ligand 6 and 16 (CXCL 6, 16) (Ma et al., 2016; Xia et al., 2013), and Galectin-3 (Gal-3) (Ionin et al., 2020; Sonmez et al., 2014; Yalcin et al., 2015)). Additional information on echo measurements, phasic volume calculations, LV/LA strain analysis and laboratory methodology can be found in the Supplemental Methods.

The data was analyzed using IBM SPSS Statistics version 23.0. The quantitative variables were expressed as mean ± standard deviation or median and interquartile range based on the normality of their distribution evaluated by the Shapiro-Wilk test. Parameters that demonstrated a non-normal distribution were logarithmically transformed for the purpose of assessing correlations and fitting linear regression models. The qualitative variables were expressed as a total number and percentage. Differences between groups were analyzed for statistical significance with the t-test, when comparing variables with normal distribution, and the Mann Whitney test for non-normally distributed variables. When comparing categorical data, contingency tables and a Chi-square or the Fisher’s exact test were used for comparison. The strength and direction of the linear relationships between pairs of continuous variables was performed using the bivariate Pearson correlation, resulting in a correlation coefficient (R).

The association of patient/echo characteristics with biomarker levels in the athlete and hypertension cohorts, respectively, was assessed through linear regression models with individual biomarker levels as the dependent variable. Firstly, each parameter was examined in univariate analysis. Correlations were checked for age, gender, BMI, BSA, systolic blood pressure, diastolic blood pressure, diabetes, dyslipidaemia, smoking status, duration of arterial hypertension and the number of hypertensive drugs (hypertensive cohort only), MET/week (athlete cohort only), basal septal thickness, LV end-diastolic diameter, LV posterior wall thickness, LV mass index, RWT, LV end-diastolic volume, LV end-systolic volume, LA/LV volume ratio, LV ejection fraction, LV 4-chamber GLS, TAPSE, septal and lateral e’ and a’ velocities, average e’ velocity, E/e’ ratio, 2D and 3D LA maximal, minimal and preP volumes, 2D and 3D LA active, passive and total emptying fraction, LA reservoir, conduit and contractile strain. When selecting variables for the multiple regression model variables with a significant zero order R correlation with the biomarker level at hand (R > 0.100) were considered. Before running the analysis, the interactions between the independent variables were assessed for correlations stronger than 0.700. In case two independent variables were highly correlated, one of them was dropped, the decision made based on strength of correlation and clinical judgement. The models were fitted using a stepwise backward variable selection. Patients were excluded based on missing values using the pairwise approach. The proportion of the variance in the dependent variable that was predictable from the independent variables was assessed using the coefficient of determination (R squared). A value of p < 0.05 was considered statistically significant.

## Results

### Exploring the patient cohort

**FIGURE 2:**
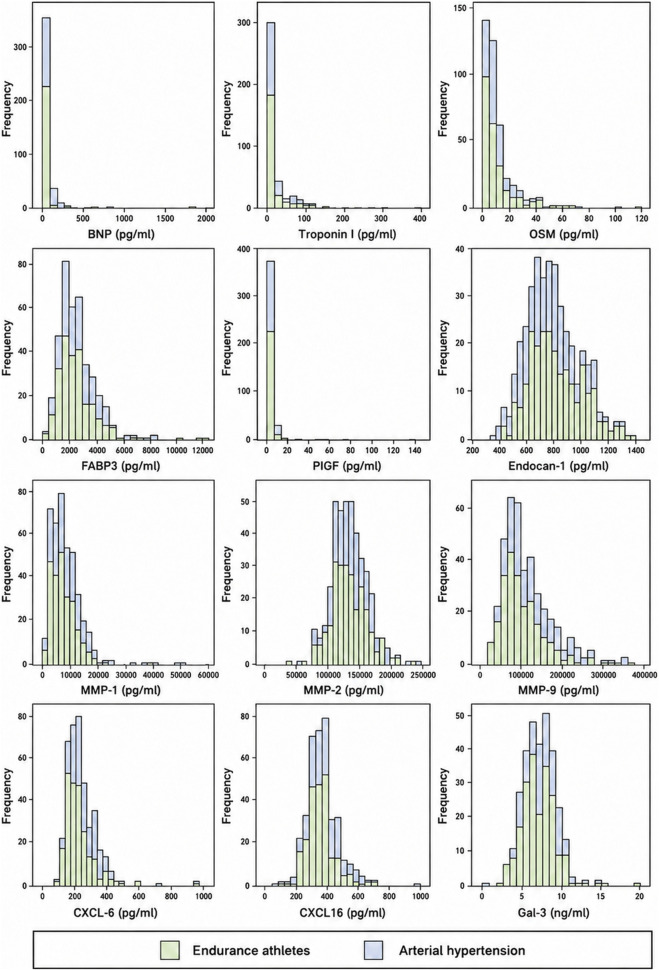
Blood biomarker range of values in different cohorts.

**FIGURE 3:**
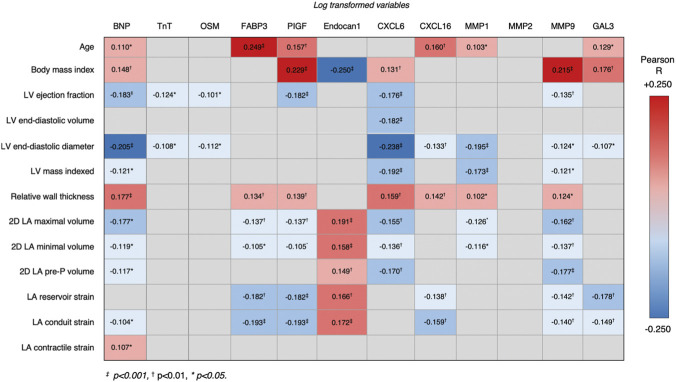
Biomarker correlations with volume and strain indices in the whole cohort–The correlation table demonstrates the complexity of variables influencing serum biomarker levels.

The comparison of patient characteristics and echocardiographic parameters can be found in the Supplementary Table S1. The comparison of biomarker levels and renal function between patient groups is shown in Table 1. The histograms in Figure 2 show the ranges of biomarker serum concentrations within the two subgroups. Markers of myocardial damage, BNP and troponin, were represented in a narrow range of values within our study, whereas OSM and FAB3 had a wider distribution of values, however without a statistical difference between subgroups. On the other hand, PIGF levels, although also narrow in range, were significantly lower in athletes as compared to hypertensive patients. Endocan-1, the biomarker related to endothelial dysfunction, was significantly higher in athletes. Finally, biomarkers of fibrosis (i.e., CXCL6, CXCL16, MMP-1, MMP-9 and Gal-3) were uniformly elevated in arterial hypertension. The estimated glomerular filtration rate was statistically lower in arterial hypertension; however, the median value was not clinically different between groups. Only FAB3 levels showed a weak correlation with impaired renal function (R −0.216, p = 0.005). Furthermore, when exploring the relationship between ACE inhibitor (n = 83, 37%) or angiotensine II receptor blocker (ARB) (n = 100, 44%) use and biomarker levels in hypertensive patients, patients on ACEi had slightly lower CXCL16 levels (p = 0.028), whereas biomarker levels did not differ based on ARB use. The correlations between biomarkers and patient characteristics, LV and LA echo measurements are shown in Figure 3 - revealing the notable influence of age and BMI on most biomarker levels. Correlations with 3D volumes is shown in Supplementary Figure S1.

**TABLE 1:** Blood biomarker levels in the patient cohorts.

Blood biomarker	Endurance training (n = 286)	Arterial hypertension (n = 225)	Group P value
Brain natriuretic peptide (BNP) (*pg/mL*)	14.3 (14.3–20.0)	20.0 (14.3–76.4)	<0.001
Troponin I (*pg/mL*)	17.0 (17.0–166.5)	59.0 (17.0–226.1)	0.211
Oncostatin M (OSM) (*pg/mL*)	7.1 (3.3–13.0)	7.9 (5.0–12.6)	0.062
Serum heart-type fatty acid-binding protein (FABP3) (*pg/mL*)	2335 (1602–3054)	2506 (1751–3464)	0.057
Placenta growth factor (PIGF) (*pg/mL*)	0.8 (0.6–2.4)	1.6 (1.0–5.0)	<0.001
Endothelial cell-specific molecule 1 (Endocan-1) (*pg/mL*)	799 (677–973)	756 (623–874)	0.002
C-X-C motif chemokine ligand 6 (CXCL6) (*pg/mL*)	198 (159–244)	236 (190–310)	<0.001
C-X-C motif chemokine ligand 16 (CXCL16) (*pg/mL*)	344 (294–388)	368 (309–446)	0.002
Matrix metalloproteinase 1 (MMP-1) (*pg/mL*)	7068 (4397–10283)	8814 (5170–12658)	0.003
Matrix metalloproteinase 2 (MMP-2) (*pg/mL*)	131148 (114835–152109)	136726 (117668–151372)	0.207
Matrix metalloproteinase 9 (MMP-9) (*pg/mL*)	91794 (66234–131278)	119423 (84670–179089)	<0.001
Galectin-3 (Gal-3) (n*g/mL*)	6.8 (5.5–8.3)	7.5 (6.0–9.0)	0.015
Estimated glomerular filtration rate (eGFR) (ml/min/1.73 m2)	90 (85–90)	90 (90–90)	<0.04

### Predicting blood biomarker levels

The univariable and multivariable regression models were fitted for the athlete and hypertensive cohorts, respectively (Tables 2–4). Considering biomarkers of myocardial damage (Table 2), the multivariable model showed that dyslipidemia predicted BNP and troponin levels in the hypertensive cohort, whereas LA active emptying fraction and average e’ mitral annulus velocity predicted troponin levels in athletes. OSM levels were related to smoking status, LV dimensions, and LA active emptying fraction in hypertension, and solely to age in athletes. Age and right ventricular longitudinal function predicted FABP3 in hypertension and athletes alike, while LA reservoir strain was significant only in the univariable model within the athlete cohort. Furthermore, BMI was associated with PIGF levels in hypertension, and LV mass and LA active emptying fraction in athletes, whereas LA contractile strain and LA maximal size were not found predictive outside univariable analysis, respectively.

**TABLE 2:** Biomarkers of myocardial damage**–**The association of patient and echo characteristics with biomarker levels in the athlete and hypertension cohorts, respectively, assessed through linear regression models with individual biomarker levels as the dependent variable (as explained in the Methods section). Abbreviations are explained in the manuscript.

Brain Natriuretic Peptide (BNP)	Arterial hypertension				Endurance athletes			
	Univariate and multiple regression predicting BNP levels in the hypertensive cohort (n=132)				Univariate and multiple regression predicting BNP levels in the athlete cohort (n=243)			
	Predictor	Pearson R	β	P	Predictor	Pearson R	β	P
	Dyslipidaemia	-0.211*	-0.191	0.028	No correlations	-	-	-
	Systolic blood pressure	-0.176*	—	-				
	3D LA passive emptying fraction	0.165*	-	-				
	R square 0.037				​			

Troponin I	Arterial hypertension				Endurance athletes			
	Univariate and multiple regression predicting troponin levels in the hypertensive cohort (n=141)				Univariate and multiple regression predicting troponin levels in the athlete cohort (n=240)			
	Predictor	Pearson R	β	P	Predictor	Pearson R	β	P
	Dyslipidaemia	-0.297*	0.108	<0.001	LV ejection fraction	-0.151*	-	-
	BSA	-0.169*	-	-	E' average velocity	-0.150*	-0.157	0.014
					2D LA active emptying fraction	-0.129*	-0.139	0.030
	​				e' lateral and e' average velocity Pearson R 0.894			
	R square 0.088				R square 0.042			

Oncostatin M (OSM)	Arterial hypertension				Endurance athletes			
	Univariate and multiple regression predicting OSM levels in the hypertensive cohort (n=170)				Univariate and multiple regression predicting OSM levels in the athlete cohort (n=243)			
	Predictor	Pearson R	β	P	Predictor	Pearson R	β	P
	Smoking status	0.157*	0.150	0.046	Age	-0.135*	-0.135	0.035
	LV end-diastolic diameter	-0.185*	-0.178	0.018	LV ejection fraction	-0.126*	-	-
	LV mass index	-0.173*	-	-				
	2D LA active emptying fraction	-0.163*	-0.150	0.046				
	LV end-diastolic diameter vs LV mass index Pearson R 0.652				​			
	R square 0.081				R square 0.018			

Serum heart-type Fatty Acid-Binding Protein (FABP3)	Arterial hypertension				Endurance athletes			
	Univariate and multiple regression predicting FABP3 levels in the hypertensive cohort (n=165)				Univariate and multiple regression predicting FABP3 levels in the athlete cohort (n=232)			
	Predictor	Pearson R	β	P	Predictor	Pearson R	β	P
	Age	0.303†	0.238	0.001	Age	0.204*	0.205	0.001
	Gender	-0.213*	-0.241	0.001	Systolic blood pressure	0.144*	-	-
	BSA	0.178*	-	-	TAPSE	-0.177*	-0.173	0.007
	Diabetes	0.190*	-	-	LA reservoir strain	-0.141*	-	-
	4C-GLS	-0.163*	-	-	2D passive emptying fraction	-0.140*	-	-
	TAPSE	-0.206*	-0.181	0.015				
	Gender and BSA Pearson R -0.567				LA reservoir strain and 2D LA passive emptying fraction R 0.521			
	R square 0.157				R square 0.076			

Placenta Growth Factor (PIGF)	Arterial hypertension				Endurance athletes			
	Univariate and multiple regression predicting PIGF levels in the hypertensive cohort (n=159)				Univariate and multiple regression predicting PIGF levels in the athlete cohort (n=239)			
	Predictor	Pearson R	β	P	Predictor	Pearson R	β	P
	BMI	-0.183*	0.171	0.032	Basal septal thickness	0.165*	-	-
	TAPSE	-0.175*	-	-	LV posterior wall thickness	0.152*	-	-
	3D LA maximal volume	-0.169*	-	-	LV mass index	0.202†	0.232	<0.001
					3D LA prep volume	0.139*	-	-
					2D LA active emptying fraction	0.179†	0.157	0.20
					3D LA active emptying fraction	0.180†	0.135	0.043
					LA contractile strain	-0.127*	-	-
	No interactions				​			
	R square 0.029				R square 0.101			
	​				Basal septal thickness vs LV mass index Pearson R 0.672			
	​				Basal septal thickness vs LV posterior wall thickness Pearson R 0.705			
	​				LV posterior wall thickness vs LV mass index Pearson R 0.625			
	​				2D LA active emptying fraction vs 3D LA active emptying fraction Pearson R 0.372			

**TABLE 3:** Biomarker of vascular endothelial dysfunction - The association of patient and echo characteristics with biomarker levels in the athlete and hypertension cohorts, respectively, assessed through linear regression models with individual biomarker levels as the dependent variable (as explained in the Methods section). Abbreviations are explained in the manuscript.

Endothelial cell-specific molecule 1 (Endocan-1)	Arterial hypertension				Endurance athletes			
	Univariate and multiple regression predicting Endocan levels in the hypertensive cohort (n=167)				Univariate and multiple regression predicting Endocan levels in the athlete cohort (n=239)			
	Predictor	Pearson R	β	P	Predictor	Pearson R	β	P
	BSA	-0.193*	-	-	BMI	-0.203†	-0.154	0.021
	BMI	-0.184*	-	-	Basal septal thickness	-0.158*	-0.143	0.037
	Systolic blood pressure	-0.206*	-0.206	0.007	3D LA maximal volume	0.147*	0.185	0.005
	BMI and BSA Pearson R 0.650				​			
	R square 0.043				R square 0.078			

**TABLE 4:** Biomarkers of fibrosis - The association of patient and echo characteristics with biomarker levels in the athlete and hypertension cohorts, respectively, assessed through linear regression models with individual biomarker levels as the dependent variable (as explained in the Methods section). Abbreviations are explained in the manuscript.

Matrix metalloproteinase 1 (MMP-1)	Arterial hypertension				Endurance athletes			
	Univariate and multiple regression predicting MMP1 levels in the hypertensive cohort (n=160)				Univariate and multiple regression predicting MMP1 levels in the athlete cohort (n=239)			
	Predictor	Pearson R	β	P	Predictor	Pearson R	β	P
	BSA	-0.208*	-0.210	0.006	LV mass index	-0.179^†^	-	-
	LV end-diastolic diameter	-0.166*	-	-	3D LA maximal volume	-0.192^†^	-	-
	e' lateral mitral annulus velocity	-0.191*	-0.200	0.009	3D LA preP volume	-0.212^†^	-	-
	3D LA active emptying fraction	0.190*	0.171	0.025	3D LA minimal volume	-0.235^†^	-0.235	<0.001
	LV end-diastolic diameter vs BSA Pearson R 0.475				3D LA maximal volume vs 3D LA PreP volume R 0.882		
	​				3D LA maximal volume vs 3D LA minimal volume R 0.838			
	​				3D LA PreP volume vs 3D LA minimal volume R 0.915			
	​				LV mass index vs 3D LA PreP volume R 0.488			
	R square 0.119				R square 0.055			

Matrix metalloproteinase 2 (MMP-2)	Arterial hypertension				Endurance athletes			
	Univariate and multiple regression predicting MMP2 levels in the hypertensive cohort (n=208)				Univariate and multiple regression predicting MMP2 levels in the athlete cohort (n=219)			
	Predictor	Pearson R	β	P	Predictor	Pearson R	β	P
	No correlations	-	-	-	MET/week	0.182*	-	-
	​				R square 0.033			

Matrix metalloproteinase 9 (MMP-9)	Arterial hypertension				Endurance athletes			
	Univariate and multiple regression predicting MMP9 levels in the hypertensive cohort (n=167)				Univariate and multiple regression predicting MMP9 levels in the athlete cohort (n=236)			
	Predictor	Pearson R	β	P	Predictor	Pearson R	β	P
	BSA	0.156*	0.234	0.002	Diastolic blood pressure	-0.160*	-	-
	Smoking status	0.165*	-	-				
	Systolic blood pressure	-0.220*	-0.261	0.001				
	2D LA active emptying fraction	-0.167*	-0.157	0.036				
	R square 0.118				R square 0.026			

C-X-C motif chemokine Ligand 6 (CXCL 6)	Arterial hypertension				Endurance athletes			
	Univariate and multiple regression predicting CXCL-6 levels in the hypertensive cohort (n=161)				Univariate and multiple regression predicting CXCL-6 levels in the athlete cohort (n=243)			
	Predictor	Pearson R	β	P	Predictor	Pearson R	β	P
	Gender	0.205^†^	0.234	0.002	Gender	0.201*	0.201	0.002
	LV end-diastolic diameter	-0.171*	-	-	LV end-diastolic volume	-0.146*	-	-
	LV end-diastolic volume	-0.198^†^	-	-	LA/LV ratio	0.132*	-	-
	LV mass index	-0.170*	-	-				
	3D LA maximal volume	-0.196*	-0.179	0.019				
	LV mass index and LV end-diastolic diameter Pearson R 0.652				LV end-diastolic volume vs LA/LV ratio Pearson R -0.415			
	LV end-diastolic diameter and LV end-diastolic diameter R 0.479				LV end-diastolic volume vs Gender Pearson R -0.493			
	R square 0.093				R square 0.041			

C-X-C motif chemokine Ligand 16 (CXCL 16)	Arterial hypertension				Endurance athletes			
	Univariate and multiple regression predicting CXCL-16 in the hypertensive cohort (n=208)				Univariate and multiple regression predicting CXCL-16 in the hypertensive cohort (n=243)			
	Predictor	Pearson R	β	P	Predictor	Pearson R	β	P
	No correlations	-	-	-	Age	0.148*	-	-
	​				R square 0.022			

Galectin-3 (Gal-3)	Arterial hypertension				Endurance athletes			
	Univariate and multiple regression predicting galectin levels in the hypertensive cohort (n=111)				Univariate and multiple regression predicting galectin levels in the athlete cohort (n=227)			
	Predictor	Pearson R	β	P	Predictor	Pearson R	β	P
	E/A	-0.238*	-0.195	0.035	BMI	0.209^†^	0.209	0.002
	LA reservoir strain	-0.286*	-0.254	0.007	2D active emptying fraction	0.134*	-	-
	LA conduit strain	-0.210*	-	-				
	LA reservoir strain vs LA conduit strain Pearson R 0.776				​			
	LA conduit strain vs E/A Pearson R 0.454				​			
	R square 0.119				R square 0.044			

Statistical Notes:* indicates statistical significance at p < 0.05. † indicates statistical significance at p < 0.01.

Abbreviations: BSA, body surface area; BMI, body mass index; LA, left atrium; LV, left ventricle; TAPSE, tricuspid annular plane systolic excursion; 4C-GLS, 4-Chamber Global Longitudinal Strain; MET, metabolic equivalent of task.

Endocan-1, the biomarker related to vascular endothelial dysfunction, was related only to systolic blood pressure in hypertensive patients, however, to BMI, LV septal wall thickness and LA maximal size in athletes (Table 3).

Regarding the biomarkers related to fibrosis (Table 4) BSA, LV longitudinal function and LA active emptying fraction predicted MMP-1 levels in hypertension, whereas, only LA minimal volume was found relevant in athletes after multivariable adjustment. No correlations were found between patient/echo characteristics and MMP-2 levels in hypertension, while the amount of METs/week predicted them within the athlete cohort. MMP-9 related to blood pressure in both cohorts, however, in hypertension it was also predicted by BSA and LA contractile function. CXCL 6 was associated with gender in both cohorts, but also LA maximal volume in hypertension, whereas CXCL 16 levels did not correlate with any characteristics besides age in athletes. Finally, Gal-3 was related to diastolic function and LA reservoir strain (R −0.286, β −0.254, p = 0.007) in hypertension, and to BMI in athletes. The main findings are summarized in the Central illustration.

## Discussion

Blood biomarkers of myocardial damage, such as BNP, serve as a direct signal of atrial remodeling in conditions such as heart failure. In our cohort, most biomarkers of myocardial damage showed a narrow range of values reflecting low prevalence of heart failure and acute myocardial damage in this relatively healthy population. Therefore, predictive models aiming to relate biomarker levels associated with myocardial damage and LA function were of limited yield (Table 1). FABP3, a protein in the cardiomyocyte cytosol released into the circulation from damaged cardiac tissue, has been associated with cardiovascular risk in the general population (Otaki et al., 2014) and with cardiovascular events in patients with arterial hypertension (Setsuta et al., 2014). Although FABP3 is not specific to atrial remodeling, levels have been shown independently associated with LA reservoir strain in athletes, presumably reflecting overall cardiac strain in the at-risk athlete cohort (Lindsay and Dunn, 2007). This relationship was also noted in our athlete’s group, however, it did not remain significant after multivariable adjustment.

Atrial fibrosis, seen in arterial hypertension (Choisy et al., 2007) and endurance training (Peritz et al., 2020), is associated with an increasing risk for atrial arrhythmias (Kottkamp, 2013; Burstein and Nattel, 2008). Circulatory biomarkers (i.e., cytokines and markers of collagen turnover, extracellular matrix remodeling, inflammation, or myocardial stretch) have been suggested to be reflective of atrial injury and fibrosis, and associated with the risk of AF. (Turagam et al., 2016). While the circulating serum levels of MMP-9 correlate with LA size (Sonmez et al., 2014), the follow-up of the Atherosclerosis Risk in Communities (ARIC) cohort demonstrated MMP-1, MMP-2 and MMP-9 levels relate to AF after adjustment for age, sex and race–with only MMP-9 levels remaining significant in the fully-adjusted model. (Huxley et al., 2013). Moreover, in hypertension, serum MMP-9 related to cardiovascular risk (Tayebjee et al., 2004). In our study, MMP-1 and 9 were found elevated in the hypertensive cohort, correlating with LA active emptying fraction, however, both levels were associated with BSA, inferring interpretation of blood levels would need to take into account patient size (Figure 3). CXCL-6 is another circulatory biomarker and a proinflammatory response chemokine associated with fibrosis. In our study CXCL-6 levels were elevated in hypertension and related to LA maximal volume, however, the levels were also influenced by sex. Finally, Gal-3, a beta-galactoside binding lectin and mediator of profibrotic pathways, has been related with LA size and fibrosis (Ionin et al., 2020; Sonmez et al., 2014; Yalcin et al., 2015) with levels predictive of AF development (Gong et al., 2020). In the 15-year follow-up of the ARIC study, plasma Gal-3 remained associated with AF risk in a multivariate model (Fashanu et al., 2017), whereas this was not seen in the 10-year follow-up of the Framingham Offspring cohort (Ho et al., 2014). Our study showed hypertensive patients had higher values of Gal-3 as compared to athletes. Moreover, Gal-3 was found independently associated with LA reservoir strain in the hypertensive cohort in the multivariable regression model, without confounding influences of patient characteristic that would complicate interpretation (i.e., gender, BMI, BSA) (Table 4; Figure 3).

The findings of this study have to be interpreted in light of the studied patient population, and the lack of biomarker specificity to atrial/myocardial remodeling. The lack of a matched control group and the underlying age and sex differences limit direct comparison between the two patient groups. However, these changes are a reflection of a real-world population characteristics, and should not bias the exploration of imaging parameters and biomarker associations within each group respectively. However, when comparing biomarker levels between different groups (e.g., as shown in Table 1) caution should be applied as biomarker levels are influenced by renal clearance and hepatic metabolism, age, sex, inflammation, and other systemic factors unrelated to atrial pathology (Forman et al., 2020) (Figure 3 and the Central illustration). Indeed, fibrosis biomarkers are not cardiac-specific but reflect systemic collagen turnover. As the LA has a relatively low tissue mass even significant atrial fibrosis might marginally impact total biomarker concentrations. Fibrosis of larger organs, such as the liver, better correlate with blood biomarkers, due to larger organ mass (Loomba and Adams, 2020). These circulating biomarkers reflect active fibrogenesis and fibrolysis whereas imaging biomarkers capture both long-term structural remodelling (such as atrial fibrosis) as well as acute conditions (such as acute changes in preload or afterload). Finally, corrections for multiple comparisons were not performed in the analysis, which increases the risk of type I error and false positive findings.

Overall, the weak correlation between circulating and imaging biomarkers reflects the complementary rather than interchangeable nature of these biomarkers. The modest relationship between gal-3 and LA reservoir strain highlights the prospective limitations of using galectin-3 as a strong predictor of LA fibrosis and remodelling, at least if used as a single parameter. Potentially, a prospective follow-up of this cohort, identifying individuals developing AF, could confirm the role of biomarker levels as another complementary screening marker of atrial cardiopathy and the risk for atrial arrhythmia development.

## Conclusion

Serum levels of fibrosis biomarkers differ between populations at risk for developing AF, although caution is needed in interpreting these levels in the context of age, sex and renal function. Gal-3 levels were found independently associated with LA reservoir function in patients with arterial hypertension, suggesting its potential role as a a biomarker related to LA fibrosis. Clinical follow-up and additional studies are needed to discern the diagnostic performance and relationship of biomarker levels and clinical outcomes.

## Data Availability

The original contributions presented in the study are included in the article/Supplementary Material, further inquiries can be directed to the corresponding author.
