# Supplementary material for: Blood biomarkers and atrial remodeling in patients at risk of atrial fibrillation
Source: Front Mol Med. 2026 May 22;6:1801523. doi: 10.3389/fmmed.2026.1801523 (PMC13235999; doi:10.3389/fmmed.2026.1801523)
Supplement: Supplementary file 1 [file DataSheet1.docx]

# DATA SUPPLEMENT

# Supplemental Methods

**Echocardiography**

LV mass was calculated by the linear method and normalized by body surface area. Relative wall thickness (RWT) was calculated by dividing the doubled value of the end-diastolic posterior wall thickness with the end-diastolic internal diameter of the LV. The ratio of LA maximal volume and LV end-diastolic volume (LV/LA ratio) was assessed to explore the relationship of LA vs. LV structural remodeling. (11) Indexed 2D and 3D phasic LA volumes (i.e. minimal, maximal, and the volume before atrial contraction at the beginning of the P wave (pre-P volume)) were used to calculate indices of LA function - total ejection fraction (related to LA reservoir function), as well as active (LA contractile function) and passive (LA conduit function) emptying fractions (10).

Pulsed-wave Doppler was performed in the apical 4-chamber view by placing the sample volume at the level of the leaflet tips to obtain mitral inflow velocities. Peak velocity of early (E) and late (A) diastolic filling, E velocity deceleration time and A wave duration were measured, and the E/A ratio calculated. Tissue Doppler was used to measure early and late diastolic mitral annular velocity at the septal (e’ and a’ septal) and lateral (e’ and a’ lateral) annular sites.

Regional myocardial deformation of the LV was assessed using speckle tracking echocardiography software on 2D grayscale images obtained from the apical 4-chamber view. The endocardial border was manually marked at end-systole of the LV. A region of interest with six segments was automatically generated. If needed, manual adjustments were performed to achieve optimal tracking. Longitudinal strain curves were generated and end-systolic strain, defined by the aortic valve closure time, was measured. LV global longitudinal strain in the 4C view was calculated by averaging values of the segments. STE analysis of LA deformation was performed in the 4- and 2-chamber apical views. Using the P wave as the onset for deformation analysis, left atrial reservoir, conduit, and contractile function were evaluated by measuring LA systolic, early diastolic and late diastolic strain, respectively. Final strain values were averaged from the 4- and 2-chamber measurements.

**Laboratory Methods**

The *Luminex Magpix* system (Luminex, Austin, TX, USA) was used to process the blood plasma and assess biomarkers. Briefly, standards or 25 µL of plasma samples and 50 µL of microparticles precoated with specific antibodies are added to each well and incubated at room temperature for 60 minutes in the dark. The plate is washed and 25 µL of detection antibody solution added, after which the plate is incubated for 30 minutes. A streptavidin-PE solution is then added, and after 30 minutes, the median relative fluorescence units from the antibody reactions measured in 120 µL of reading buffer using a *Luminex 200 analyzer* and the *xPONENT software* (v. 3.1; Luminex, Austin, TX, USA). The concentration of each analyte is calculated using five-parameter regression models. Only the standard points with recoveries ranging from 70 to 130% are considered. The intra-assay coefficient of variation was less than 8%. In individual samples with values at the detection level, the lower or the higher level of detection was used in the calculation of the results.

**Statistical analysis**

When performing linear regression scatter plots were visually inspected for each variable to determine linearity of the dependent/independent variable relationships. Homoscedasticity was assessed by plotting the regression standardized residual vs the predictive value, whereas normality of the residuals with a Q-Q plot.

**Supplementary results**

Endurance athletes performed training sessions corresponding to a median of 5716 (4112-7640) MET minutes/week. The hypertensive cohort had an average duration of 8 (5-15) years since initial diagnosis, and an average of 1.3 ± 0.6 prescribed antihypertensive medications. Qualitative assessment in the MR cohort classified regurgitation as moderate in 35% (n=13) and severe in 65% (n=24) of patients.

The respective cohort characteristics are shown in **Table 1**. Endurance athletes were significantly younger and predominantly male, as compared to the hypertensive and MR subgroups. Hypertensive patients had higher systolic blood pressure preceding the echo examination, a significantly higher prevalence of comorbidities, a worse lipid profile, and an overall higher use of antihypertensive, diuretic or lipid lowering drugs. The hypertensive LV was smaller in end-diastolic volume with clear signs of hypertrophy (based on wall thickness and relative wall thickness (RWT)). In comparison, athletes and MR patients had a significantly larger ventricle and LV mass, but with a lower RWT. In athletes, the MET minutes/week correlated positively with LV end-diastolic volume (Pearson R 0.251, p=0.001) and LV mass index (Pearson R 0.317, p<0.001). Furthermore, in the MR cohort, severe MR was associated with a higher LV mass index as compared to moderate MR (102 ± 20 vs 84 ± 19, p=0.011). Duration of hypertension or the number of prescribed antihypertensive drugs did not correlate with parameters of LV structure. The mitral E/A ratio was highest in the hypertensive group, whereas PW TDI septal and lateral e’ annular velocities were significantly higher in athletes, and a’ velocities lower. Global function showed reduced ejection fraction in hypertension, as compared to the remaining subgroups, however, 4C-LV global longitudinal strain (GLS) was lowest in the athlete cohort.

In comparison with athletes and MR patients, the hypertensive patients had the smallest LA maximal, minimal and pre-P size, in both 2D and 3D measurements, and the lowest percentage of individuals with an 2D indexed maximal LA size of more than 34 ml/m^2^. The LA reservoir function - 3D LA ejection fraction and LA reservoir strain - was higher in the athletes, reduced in hypertension, and lowest in MR patients. A similar finding was seen in LA conduit function, where the 3D passive emptying fraction and the LA conduit strain were the highest in athletes, and significantly reduced in the rest of the cohort. Finally, LA contractile function, as reflected by 2D and 3D active emptying fractions and LA contractile strain, was accentuated in hypertension. In the athlete cohort, the MET minutes/week demonstrated a positive correlation with LA volumes, strongest seen in relation to pre-P LA volume (Pearson R 0.296, p<0.001), and a weak negative correlation with LA reservoir (Pearson R 0.127, p=0.047) and conduit strain (Pearson R -0.132, p=0.038). In the MR cohort, severe MR was related to larger LA maximal volumes as compared to moderate MR (48 ± 14 vs. 37 ± 7 ml/m^2^, p=0.001), however no differences were seen in volumetric indices or STE deformation. While duration of hypertension did not correlate with LA parameters, the number of prescribed antihypertensive drugs weakly correlated with LA size, most strongly with the pre-P volume (Pearson R 0.184, p=0.007), and inversely with LA reservoir (Pearson R, -0.166, p=0.014).

**Supplementary Results**

**Supplementary Figures**

**Figure S1 Biomarker correlations with 3D volume and strain indices** – Correlation of 3D LA volumes reflected the correlations seen with 2D volumes.

**
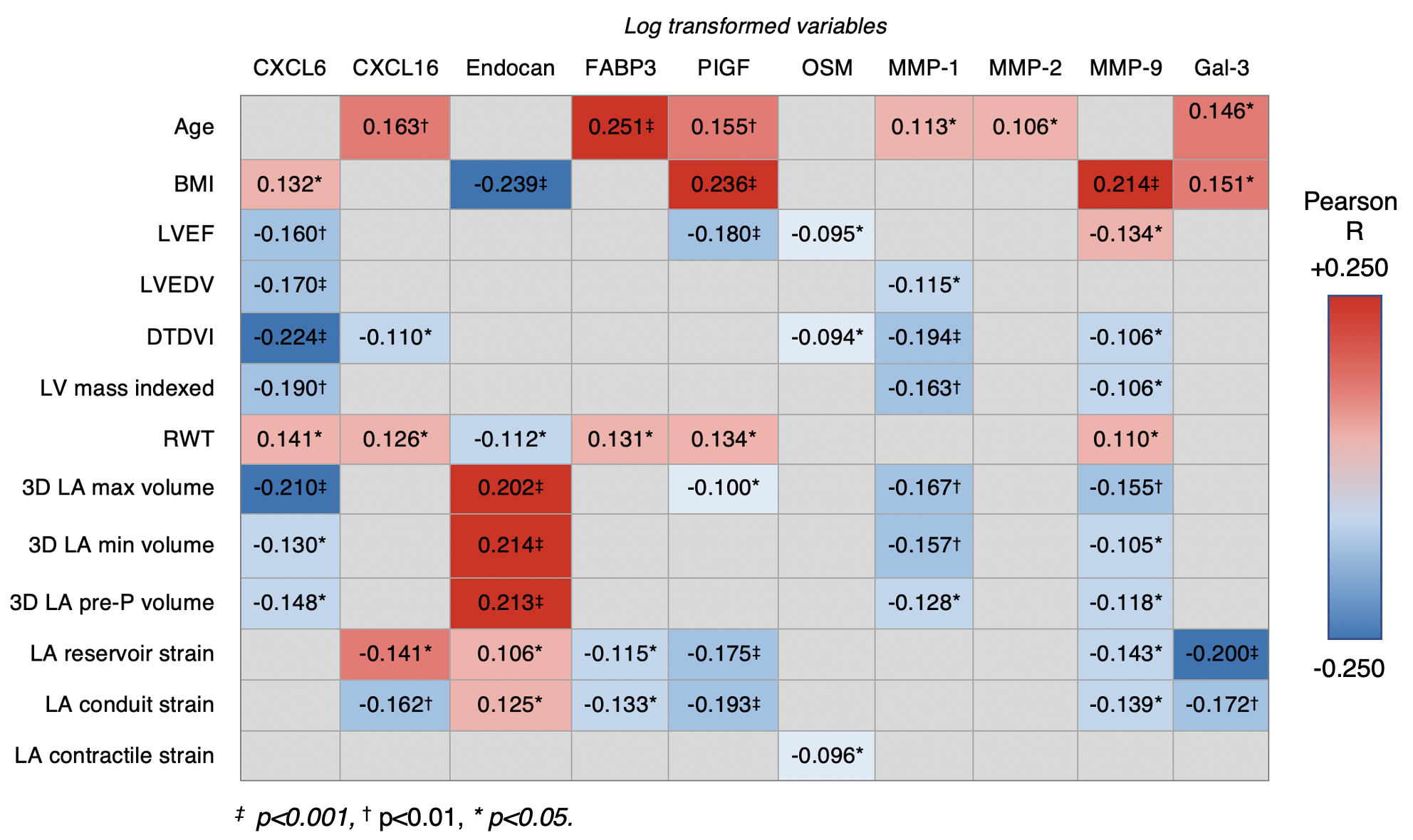
**

**Supplementary Tables**

**Table S1** General cohort characteristics

| Variables | | Endurance training  (n=286) | Arterial Hypertension  (n=225) | Group P value |
| --- | --- | --- | --- | --- |
| Age (years) | | 47 (44-51) | 58 (53-62) | <0.001 |
| Men (%) | | 235 (82%) | 125 (56%) | <0.001 |
| Body mass index (kg/m2) | | 23.4 (21.8-24.8) | 27.4 (24.8-31.2) | <0.001 |
| Body surface area (m^2^) | | 1.9±0.2 | 1.8±0.4 | <0.001 |
| Systolic blood pressure (mmHg) | | 124 (116-132) | 136 (127-147) | <0.001 |
| Diastolic blood pressure (mmHg) | | 73±9 | 81±10 | <0.001 |
| Heart rate (beats per minute) | | 52 (47-56) | 68 (61-75) | <0.001 |
| Diabetes mellitus (%) | | 1 (1%) | 21 (9%) | <0.001 |
| Dyslipidemia (%) | | 70 (28%) | 105 (54%) | <0.001 |
| Smoking (current or former) (%) | | 59 (21%) | 93 (42%) | <0.001 |
| Beta-blockers (%) | | 0 | 43 (20%)) | <0.001 |
| ACE inhibitors (%) | | 7 (3%) | 83 (38%) | <0.001 |
| Angiotensin Receptor Blockers (%) | | 0 | 100 (46%) | <0.001 |
| Calcium channel blockers (%) | | 1 (1%) | 62 (28%) | <0.001 |
| Diuretics (%) | | 2 (1%) | 80 (36%) | <0.001 |
| Statins (%) | | 3 (1%) | 50 (23%) | <0.001 |
| Other lipid lowering drugs (%) | | 6 (2%) | 31 (14%) | <0.001 |
| LV ejection fraction (%) | | 64 (59-68) | 58 (54-63) | <0.001 |
| LV global longitudinal strain (%) | | 19.98±2.28 | 20.80±2.73 | <0.001 |
| LV end-systolic volume (ml) | | 39 (31-48) | 41 (31-51) | 0.177 |
| LV end-diastolic volume (ml) | | 109±26 | 100±29 | <0.001 |
| Basal inferoseptal wall thickness (mm) | | 11 (10-11) | 12 (11-13) | <0.001 |
| LV internal diameter at end-diastole (mm) | | 47.8±4.6 | 42.6±6.4 | <0.001 |
| Posterior wall thickness (mm) | | 10 (10-11) | 11 (10-12) | 0.43 |
| Indexed LV mass (g/m2) | | 97 (86-110) | 85 (74-98) | <0.001 |
| Relative wall thickness | | 0.44 (0.40-0.48) | 0.51 (0.44-0.60) | <0.001 |
| E velocity (cm/s) | | 73 (64-81) | 71 (62-82) | 0.520 |
| A velocity (cm/s) | | 53 (48-62) | 74 (64-87) | <0.001 |
| E/A ratio | | 1.3 (1.1-1.5) | 0.9 (0.8-1.2) | <0.001 |
| Mitral annulus septal e’ velocity (cm/s) | | 11 (9-12) | 8 (7-9) | <0.001 |
| Mitral annulus lateral e’ velocity (cm/s) | | 14 (13-17) | 10 (8-12) | <0.001 |
| Mitral annulus septal a’ velocity (cm/s) | | 9 (8-10) | 10 (9-11) | <0.001 |
| Mitral annulus lateral a’ velocity (cm/s) | | 9 (8-10) | 11 (9-13) | <0.001 |
| E/e’ | | 5.7 (5.0-6.6) | 8.1 (6.7-9.6) | <0.001 |
| Tricuspid annular plane systolic excursion (mm) | | 24.5±4.2 | 22.2±3.8 | <0.001 |
| LA maximal volume (ml/m^2^) | 2D | 41±9 | 30±8 | <0.001 |
|  | 3D | 44 (38-51) | 33 (27-38) | <0.001 |
| LA maximal volume > 34 ml/m^2^ | 2D | 224 (78%) | 55 (25%) | <0.001 |
| LA/LV maximal volume ratio | 2D | 0.69 (0.60-0.81) | 0.56 (0.46-0.67) | <0.001 |
| LA minimal volume, (ml/m^2^) | 2D | 18 (15-22) | 12 (10-16) | <0.001 |
|  | 3D | 19 (16-22) | 15 (12-18) | <0.001 |
| LA pre-atrial contraction volume, (ml/m^2^) | 2D | 29 (24-34) | 21 (17-26) | <0.001 |
|  | 3D | 28 (25-32) | 22 (18-26) | <0.001 |
| LA reservoir strain (%) | | 34.17 ± 4.26 | 29.32 ± 5.25 | <0.001 |
| LA ejection fraction (%) | 2D | 55±7 | 55±9 | 0.415 |
|  | 3D | 58 (54-61) | 55 (49-60) | <0.001 |
| LA conduit strain (%) | | 19.32±4.16 | 13.70±4.53 | <0.001 |
| LA passive emptying fraction (%) | 2D | 28±8 | 27±9 | 0.036 |
|  | 3D | 37 (33-42) | 32 (24-39) | <0.001 |
| LA contractile strain (%) | | 15.07 (13.41-16.31) | 15.61 (13.21-17.95) | <0.001 |
| LA active emptying fraction (%) | 2D | 36 (31-41) | 39 (33-45) | 0.006 |
|  | 3D | 32±7 | 33±10 | 0.026 |
| LA contractile/conduit strain ratio | | 0.78 (0.63-0.93) | 1.14 (0.88-1.46) | <0.001 |
| BNP (*pg/ml*) | | 14.3 (14.3-20.0) | 20.0 (14.3-76.4) | <0.001 |
| CXCL6 (*pg/ml*) | | 198 (159-244) | 236 (189-310) | <0.001 |
| CXCL16 (*pg/ml*) | | 344 (294-388) | 368 (309-446) | 0.002 |
| Endocan-1 (*pg/ml*) | | 799 (677-973) | 756 (623-874) | 0.002 |
| FABP3 (*pg/ml*) | | 2335 (1602-3054) | 2506 (1751-3464) | 0.057 |
| PIGF (*pg/ml*) | | 0.8 (0.6-2.4) | 1.6 (1.0-5.0) | <0.001 |
| OSM (*pg/ml*) | | 7.1 (3.3-13.0) | 7.9 (5.0-12.6) | 0.062 |
| Troponin I (*pg/ml*) | | 17.0 (17.0-166.5) | 59.0 (17.0-226.1) | 0.211 |
| MMP-1 (*pg/ml*) | | 7068 (4397-10283) | 8814 (5170-12658) | 0.003 |
| MMP-2 (*pg/ml*) | | 131148 (114835-152109) | 136726 (117668-151372) | 0.207 |
| MMP-9 (*pg/ml*) | | 91794 (66234-131278) | 119423 (84670-179089) | <0.001 |
| Gal-3 (n*g/ml*) | | 6.8 (5.5-8.3) | 7.5 (6.0-9.0) | 0.015 |
| Hemoglobin (*g/L*) | | 145 (138-151) | 145 (135-154) | 0.847 |
| Glomerular filtration (*ml/min/1.73m^2^*^)^ | | 90 (90-90) | 90 (85-90) | 0.002 |
| Total cholesterol (*mg/dl*) | | 194±30 | 200±29 | 0.034 |
| LDL cholesterol (*mg/dl*) | | 113±28 | 122±27 | 0.003 |
| HDL cholesterol (*mg/dl*) | | 61 (53-72) | 49 (41-58) | <0.001 |
| Triglycerides (*mg/dl*) | | 74 (56-100) | 131 (96-193) | <0.001 |
